# Applying Q-Methodology to Investigate People’ Preferences for Multivariate Stimuli

**DOI:** 10.3389/fpsyg.2020.556509

**Published:** 2020-12-10

**Authors:** Jie Gao, Alessandro Soranzo

**Affiliations:** ^1^Institute of Education, University College London, London, United Kingdom; ^2^Department of Psychology, Sociology and Politics, Sheffield Hallam University, Sheffield, United Kingdom

**Keywords:** individual differences, dominance – rank orders, preference, repeated measures analyses, Q-methodology

## Abstract

This article serves as a step-by-step guide of a new application of Q-methodology to investigate people’s preferences for multivariate stimuli. Q-methodology has been widely applied in fields such as sociology, education and political sciences but, despite its numerous advantages, it has not yet gained much attention from experimental psychologists. This may be due to the fact that psychologists examining preferences, often adopt stimuli resulting from a combination of characteristics from multiple variables, and in repeated measure designs. At present, Q methodology has not been adapted to accommodate. We therefore developed a novel analysis procedure allowing Q-methodology to handle these conditions. We propose a protocol requiring five analyses of a decision process to estimate: (1) the preference of stimuli, (2) the dominance of variables, (3) the individual differences, (4) the interaction between individual differences and preference, and (5) the interaction between individual differences and dominance. The guide comes with a script developed in R (R Core Team, 2020) to run the five analyses; furthermore, we provide a case study with a detailed description of the procedure and corresponding results. This guide is particularly beneficial to conduct and analyze experiments in any research on people’s preferences, such as experimental aesthetics, prototype testing, visual perception (e.g., judgments of similarity/dissimilarity to a model), etc.

## Introduction

Decision-making is a very complex process during which individuals draw on different resources to inform their choices (Weber and Johnson, 2009). Psychologists have developed a number of theoretical models to interpret decisions (Guitoni and Martel, 1998). This article seeks to contribute to the psychological inquiry into decision-making in relation to preferences (i.e., aesthetic preferences) by extending the application of Q-methodology and by further developing its data analysis capabilities.

Developed by William Stephenson in the 1930s (Stephenson, 1935a,b) Q-methodology was originally designed to investigate subjectivity (e.g., attitudes, viewpoints, perspectives, and so on). It provides a robust and systematic method to reveal consensus and disagreement among responders. While Q-methodology has been widely applied in fields such as sociology (e.g., Hedges, 2014), education (e.g., Gao, 2019) and political sciences (e.g., Lehtonen and Aalto, 2016) it has not yet gained much attention from psychologists interested in decision-making, despite of its methodological advantages that are summarized below. This may be partly due to the fact that conventional Q-methodology studies mostly use statements (with some exceptions, e.g., Gauger and Wyckoff, 1973; Gelineau, 1981; Somerstein, 2014), whereas in psychologists studding preferences usually adopt stimuli such as images, sounds, etc. Furthermore, these stimuli often include a combination of characteristics from multiple variables and they are presented, in repeated measures designs.

To our knowledge, there is no comprehensive guidance on how to conduct a Q-methodology study with stimuli combining characteristics from multiple variables and how to comprehensively analyze the data collected with this procedure. While psychologists usually measure the stimulus characteristics most identified by participants, the conventional analysis in Q-methodology does not typically pay much attention to this aspect. For example, in experimental aesthetics - the field of psychology funded by Fechner (1860) pertaining to the empirical investigation of the sensations evoked by stimuli – psychologists are chiefly concerned with the characteristics of a stimulus which are perceived *overall* as beautiful or appealing. This article provides a detailed account on how to analyze this data in Q-methodology; thereby promoting the application of Q-methodology in experimental psychology.

This article is organized in four sections. Section one outlines the advantages of Q-methodology. Section two explains why it is meaningful to distinguish between preference and dominance of the variables in study. Section three demonstrates the procedure of measuring dominance (i.e., the relative importance of a variable). Section four provides a comprehensive protocol to conduct the analysis and interpret the data with an example study.

## Advantages of Q-Methodology

Many methods to measure Multi-Criteria Decision-Making methods (MCDM) have been proposed; a comprehensive review is presented by Guitoni and Martel (1998). As the authors outlined, none of these methods can be considered optimal for all kind of experiments as they have been developed for specific purposes. For example, the SMART and SMARTS and SMARTER (Edwards, 1977; Edwards and Barron, 1994) which base their analysis on a weight attribution to ratings (or raking in the case of SMARTER) have been specifically developed for market research. Although these methods of analysis could be applied to experimental psychology, they present a number of limitations (see Guitoni and Martel, 1998; Table 1) that restrain their use. For example, elegant methods such as the UTA method (Jacquet-Lagreze and Siskos, 1982) that consider utility functions, have been successfully applied in managerial decision-making, but it might not be appropriate to process ordinal data such as those arising from a study on preferences.

**TABLE 1:** Results of Wald chi-square test.

**Variable**	***df***	***Chi*-square**	***p***
Size	1	2.05	0.15
Texture	1	6.44	0.004
Contour	1	10.38	0.001
Behavior	3	206.74	<0.001

**FIGURE 1:**
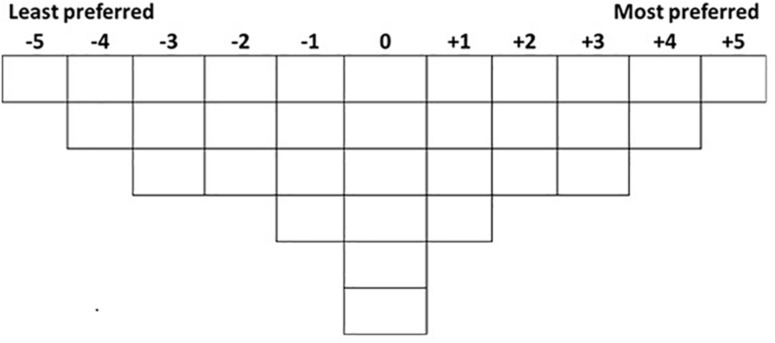
Example of a Q-sorting grid for an experiment with 32 stimuli.

We advanced a new MCDM specifically devoted to experimental aesthetics which benefits of the advantages of Q-methodology. Firstly, it has a unique data collection method, the Q-sorting procedure, which has several appealing characteristics. In the Q-sorting procedure, participants are required to rank-order a set of stimuli (or statements) into one single continuum based on instruction. The shape of the continuum is quasi-normal (in the sense that it resembles a normal gaussian distribution with more stimuli in the middle than in the tails of the continuum) to form a bell-shaped grid (Brown, 1980)^1^. Figure 1 shows an example of a Q-sorting grid for 32 stimuli. The figure shows a “typical” configuration for an experiment with 32 stimuli; however, this shape could be altered if desired. Brown (1980) showed that the actual shape of the grid does not affect the analysis of the results.

The Q-sorting procedure is less time-consuming, more engaging for participants and more natural than assigning abstract scores using questionnaires with Likert scales (Klooster et al., 2008). Participants are given the opportunity to make a systematic comparison between all the items presented at the same time. Therefore, the Q-sorting outcome is based on a holistic thinking process rather than isolated ratings (Watts and Stenner, 2012). The Q-sorting procedure facilitates the decision process and enables participants to accurately differentiate the subtle differences in their judgments. Moreover, the Q-sorting procedure minimizes the order effect, which psychologists often encounter in experiments (Atmanspacher and Römer, 2012). As all stimuli are presented simultaneously, participants can change the relative rank of each stimulus in the continuum, making therefore possible comparisons among all stimuli. Hence, as order and fatigue effects are minimized and there is the possibility to change the ranking, a more coherent and accurate analysis of the decision process can be obtained.

In Q-methodology studies, Q-factor analysis identifies the clusters of participants who produce similar outcomes. The clustering of participants instead of items differentiates this from a conventional factor analysis. This statistical procedure is similar to Latent Profile Analysis (Vermunt and Magidson, 2002) and clustering analysis (Kaufman and Rousseeuw, 2009), which all serve the purpose of grouping similar participants into clusters. The identification of clusters of participants can be appealing to experimental psychologists who are interested in individual differences. Traditional experimental designs that aim to investigate individual differences often build upon the assumptions that these differences result from demographic or personality traits. Q-methodology instead takes a bottom-up approach to reveal individual differences beyond any prior assumptions. The Q-factor analysis of Q-sorting data generates evidence of individual differences based on the data *per se* (Zabala and Pascual, 2016). Based on the emerged clusters of participants, psychologists can further explore where the individual differences come from. For example, by examining whether participants’ gender is differently distributed across the Q-factors, a researcher can infer whether gender differences exist.

Furthermore, psychologists may want to explore individual differences even when this is not their primary goal. Appelt et al. (2011) underlined that researchers should not neglect the effects of individual differences. In situations where no clear tendency emerges from the overall sample, it is likely that individual differences have canceled out the expected effect and that the effect was evident but only for a sub-set of participants. For example, in experimental aesthetics, scholars found stable and statistically robust individual preferences which were masked by weak population preference (McManus, 1980; McManus et al., 2010). Q-methodology enables psychologists to detect potential individual differences.

In addition, taking a mixed-methods approach, Q-methodology also includes a post-sorting interview which allows the rankings to be interpreted qualitatively (Watts and Stenner, 2012). Interview data complements the Q-sorting data to provide a more detailed and comprehensive account of the data than other methods.

## Preference vs. Dominance

We introduce a new data analysis procedure which will add to these methodological advantages, extending the application of Q-methodology to experimental psychology.

People pay attention to multiple information when making decisions. For example, when judging the aesthetics of a painting, people may consider variables such as the style, the compositions, the colors, etc., at the same time.

People may differ in their *preference* for a certain variable but agree on the importance of the variable. We refer to the importance of a variable as its *dominance*. For example, individual A may prefer contemporary art whilst B prefers pop art; but both A and B may regard the style as the dominant variable, the variable on which they put most of the focus for their choice. Similarly, in a study on facial attraction, the researcher might wish to examine preferred eye color or shape of mouth, as well as which of these two variables is the most dominant in determining facial attraction. To recap, we use the term “*preference*” to indicate the preference for one level over another *within* the same variable (e.g., preference for blue eyes over green eyes), and the term “*dominance*” to indicate the importance of a variable *across* the variables (e.g., dominance of eye color over mouth shape).

By supplementing conventional Q-methodology with the possibility of investigating *dominance* and distinguishing it from *preference*, we provide a more comprehensive inquiry to deepen the study of decision making in experimental psychology.

Distinguishing between dominance and preference has an additional advantage. In conventional Q-methodology these two aspects are intertwined with each other and the clusters of people emerging from the Q-factor analysis represent a combination of both aspects. Hence, participants may differ in both preference and dominance, or on just one of them without this explicitly emerging from the analysis. By differentiating between these two aspects of the decision process, it is possible to measure the interactions between Q-factors and *both* preference and dominance. This is meaningful because it allows clarifying the similarities among people to be identified as well as the differences.

In this way, it is possible to conduct a more comprehensive analysis. In particular, we propose a protocol consisting of five analyses each aimed at answering to a specific research question:

(1) Analysis of overall preference: Which are the overall preferred characteristics of each variable?
(2) Analysis of overall dominance: Which are the important variables that influence people’s decisions?
(3) Analysis of individual differences: Do people differ in their decisions?
(4) Analysis of the interaction between individual differences and preferences: Do different clusters of people prefer different characteristics of a variable?
(5) Analysis of the interaction between individual differences and dominance: Are different clusters of people driven by different variables?

## Importance of a Variable: The Qdominance () Function

The QDominance()function provided in the supplemented QDominance.R file is designed to measure the dominance of each variable. The analysis of dominance has a similar meaning in a regression analysis of finding out whether a variable can predict an outcome. QDominance() utilizes the Q-sorting data directly and provides a more straightforward way to address the issue of the importance of a variable.

The dominance (D) of each variable (_v_) is given by the maximum difference between the sums of scores of each level (l) in a variable with *n* levels (equation 1):

(1) $$D_{v}=M\,a\,x\,\left(C\,\left(\sum_{i=1}^{i=n}\left(l_{i}\right)-\sum_{i=1}^{i=n}\left(l_{=i}\right)\right),2\right)$$

This is the maximum value resulting from the combinations of two of the differences between the sums of scores of the stimuli in the same level with those of the stimuli in the other levels. Intuitively, the dominance of a variable is a measure of the spread of the stimuli across the Q-sorting grid based on its levels. If a variable is very important, all the stimuli or items in the same level that share a desirable characteristic will receive a high score whilst the stimuli which do not possess that specific desirable characteristic, will all receive a low score. The difference between the scores will be relatively large in this case. Vice-versa if a variable is not important, the stimuli in the same level will be scattered over the grid rather than receiving extreme scores, making the difference between the sums relatively small.

Equation 1, however, cannot be used directly to compare different variables in experimental designs where variables have different number of levels. This is because of the nature of the Q-grid; the smaller the number of the level of a variable is, the higher the maximum difference will be. That is, the maximum difference between the levels of a variable depends on the number of its levels. For example, for a variable with only two levels the maximum difference that would be obtained would be when all the stimuli in one level (i.e., half of the experimental stimuli) are highly ranked whilst the stimuli in the other level (i.e., the other half of the experimental stimuli) are ranked lowly. If instead a variable has 4 levels, the maximum difference that can be obtained would be when only 1/4 of the experimental stimuli are highly ranked and 1/4 are lowly ranked. Variables with a lower number of levels therefore have a higher potential maximum difference. For this reason, Dv has to be weighted for the maximum difference that each variable can get, which depends on the number of its levels *n*. Equation 2 shows how to calculate the weighted dominance for variable v (WD_v_).

(2) $$W\,D_{v}=\frac{D_{v}}{\left(\sum_{i=\frac{n\,s\,t\,i\,m\,u\,l\,i}{n_{v}}}^{i=\max\,\mathrm{ingrid}}s\,c\,o\,r\,e\,i\,n\,g\,r\,i\,d_{i}\right)}\times 2$$

The maximum difference for a certain variable (the denominator in equation 2) is twice the maximum the sum of the rating scores of stimuli (or items) at the same level. This is the sum of each score in the Q-sort grid (scoreingrid) from the maximum value in the grid (maxingrid; +5 in the example in Figure 1) backward to the number of items or stimuli (nstimuli) divided by the number of levels in the variable v (n_v_).

The product between the weighted scores and the average of the maximum differences between the v variables gives the Comparable Score Difference for each variable (CSDv). This difference among the scores is comparable across all variables (equation 3).

(3) $$C\,S\,D_{v}=W\,D_{v}\times \left(\frac{\sum_{i=\frac{n\,s\,t\,i\,m\,u\,l\,i}{n_{v}}}^{i=m\,a\,x\,i\,n\,g\,r\,i\,d}s\,c\,o\,r\,e\,i\,n\,g\,r\,i\,d_{i}}{N}\right)\times 2$$

It is useful to get the proportion of dominance for each variable (PD_v_). This is the weighted average of the weighted dominance (WD_v_), shown in equation 4:

(4) $$P\,D_{v}=\frac{W\,D_{v}}{\sum_{i=1}^{i=N}W\,D_{v_{i}}}$$

These equations are implemented in the supplemented QDominance() function. This function takes three arguments as input: 1) scores: a vector of scores or a vector/matrix of Q-factor scores; 2) v: A matrix indicating the levels for each variable; and 3) isfs: logical value: if this is set to True, scores are Q-factor scores, generated by the Q-factor analysis (see below Analysis 3); if False, they are scores from the Q grid; that is the ranks provided by all the participants (default is False).

QDominance() returns the Proportion of Dominance (PD) for each variable v if isfs is False; and it returns both the Comparable Score Difference (CSD) as well as the Proportion of Dominance (PD) for each variable v if isfs is True.

By incorporating QDominance() in the original Q-methodology, this procedure can be used to address a wide range of research questions in the decision process; specifically, the five analysis outlined above. The following section presents a detailed guide of how to use the protocol. An example with simulated data is provided to help readers gain a better understanding of the method.

## Protocol

To conduct a Q-methodology study, the first step is to design the Q-sorting grid and prepare the stimuli according to the research question. Readers seeking detailed instruction on how to prepare for Q-sorting can refer to Brown (1980). To demonstrate how the protocol works, we present a study example on Interactive Objects (IOs) which are objects which contain electronic components that exhibit autonomous behavior when handled, e.g., vibrating, playing a sound, or lighting-up. This is a repetition of a study conducted by Soranzo et al. (2018) which used a the following method. In a first qualitative phase, the aesthetic dimensions to be measured were identified. In a second, experimental phase, participants rated each object on a scale of 1 to 7 on each of the dimensions emerged in the first phase. The study overall included a large number of participants (more than 600) and one variable was measured between subjects. In this study, instead, only eighteen participants were included, and all the variables were measured within subjects. This small number of participants was decided to examine whether similar results obtained with a large sample size can be replicated with a smaller one by adopting the Q-methodology. A small number of participants is typical in Q methodology (Brown, 1980). Participants were provided with thirty-two IOs with different levels of four variables: 1) Size, 2) Surface texture, 3) Contour and 4) Behavior. The variables differ in the number of levels (this illustrates the procedure properly) as indicate in Table 2. Participants were asked to rank-order these IOs into the quasi-normal Q-grid.

**TABLE 2:** The variables with the corresponding levels of the study example.

**Form**			**Behavior**
***Size***	***Surface texture***	**Contour**	
Small (7.5 cm)	Smooth (plastic)	Round (sphere)	Emit a light
Large (15 cm)	Rough (fabric)	Angular (cube)	Play a sound
			Vibrate
			Quiescent

In line with Watts and Stenner’s guidance (Watts and Stenner, 2012), with a total of thirty-two stimuli, a grid such as the one in Figure 1, with ranks ranging from −5 (’the least preferred’) to +5 (’the most preferred), is appropriate as it provides adequate variety in the rankings, yet it does not over-complicate the procedure.

## Data Preparation

After the experiment has been conducted, the data needs to be available in a tabular/matrix format and saved as *data.csv* file. The Q-sorting data are organized in a matrix with stimuli in rows and participants in columns. In addition to the data file, users also need to generate a spreadsheet indicating the levels of variables for each of their stimulus (the *variables.csv* file). The variable file is organized as stimuli in rows and variables in columns. Each row indicates the levels of each variable in the dataset. Levels can be entered as names or numbers.

The dataset (*data.csv)* and the variable file (*variable.csv*) of the IOs study are included in the Supplementary Materials for download.

## R Scripts

There are two R files: the Qdominance.R which runs the Qdominance() function and the QmultiProtocol.R which conducts the five analysis outlined above. A step-by-step tutorial of applying the QmultiProtocol.R is also included in the Supplementary Materials. The complete project (i.e., R scripts and data examples) is available online^2^.

The QmultiProtocol.R makes use of the following packages:

’qmethod’ (Zabala, 2014);

’ordinal’ (Christensen, 2018);

’data.table’ (Dowle et al., 2019).

Users need to store both R scripts in the same working directory with the two ^∗^*.csv* spreadsheets (i.e., the dataset and the variable file). The protocol can be readily used without further programming (if not wished) apart from setting the working directory’s path in Line 1 by the setwd()function and specifying the number of Q-factors in Line 3. The QmultiProtocol.R generates a text file named *Qfact.txt* which contains the information needed to decide how many Q-factors to select (see Analysis 3 for suggestions on how to decide the number of Q-factors). The *Qfact.txt* is automatically saved in the same working directory as specified by the user.

## Analysis 1: Overall Preference: Which Are the Overall Preferred Characteristics of Each Variable?

**FIGURE 2:**
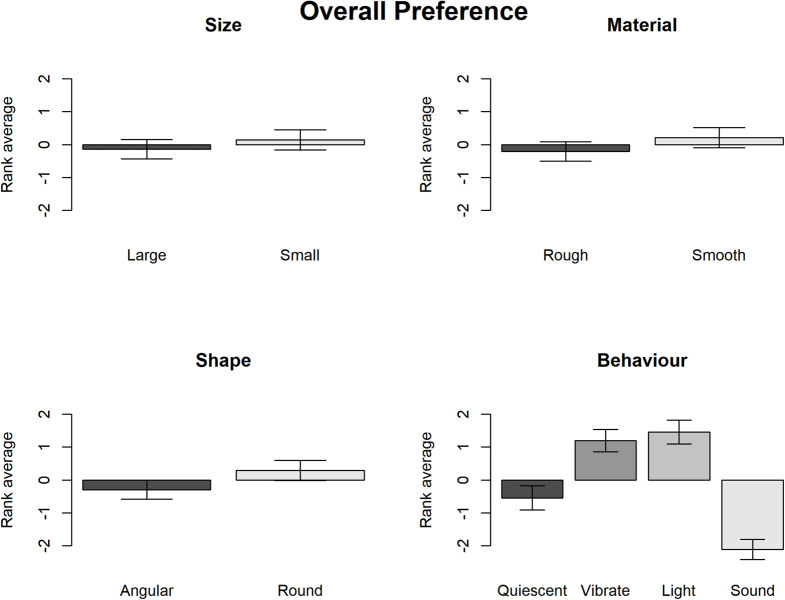
The rank of overall preference for each variable of the study example.

The first analysis aims to answer the question of whether people share similar preferences, for example, whether overall the participants prefer Level 1 to Level 2 of Variable A. Q-sorting data is distributed at an ordinal level and data are entangled and interdependent because there is a fixed number available for each rank (see Figures 1, 2). Therefore, data need to be analyzed with an ordered-probit model (Liddell and Kruschke, 2018). Moreover, Q-sorting data have an additional characteristic: the median and deviance of the ranks are the same for all subjects. Hence, although data come from a within-subject procedure, there is no subject-level variation, i.e., no random effects.

The QmultiProtocol.R adopts the frequentist approach and runs the ordered-probit model by the clm() function included in the “ordinal” package (Christensen, 2018). The effects of the independent variables are tested by analyzing the deviance, which is analogous to analyzing the variance in linear models.

Model assumptions are tested by the nominal_test() and the scale_test() functions; which provide the likelihood ratio test of the proportional odds assumption and of the scale effect assumption, respectively.

Lines 19–109 in *QmultiProtocol.R* perform Analysis 1. The clm() function is used to examine the cumulative link model for the overall preference. As for the present IOs study example, the estimates and corresponding z scores of each variable are illustrated in Table 3.

**TABLE 3:** The variable estimates of the study example.

	***Estimate***	***Z***	***p***
SizeSmall	0.21	1.43	0.152
TextureSmooth	0.37	2.47	0.013
ContourRound	0.48	3.22	0.001
BehaviourVibrate	1.48	6.91	<0.001
BehaviourLight	1.75	7.96	<0.001
BehaviourSound	−1.40	−6.42	<0.001

The nominal test reveals that there is no violation of the partial proportional odds (all *ps* > 0.02) whilst there is a small violation of the scale assumption for the Surface texture variable (LRT(1) = 6.09; *p* = 0.014). Therefore, the results from this variable should be interpreted with caution.

Subsequently, the Wald chi-square test shows that participants demonstrated relatively strong overall preferences in terms of the Surface texture, Contour and Behavior but not the Size (see Table 1). The graphs in Figure 2 show that overall participants preferred smooth to rough surface texture, round to angular shape and lighting/vibrating to sounding/quiescent objects.

## Analysis 2: Overall Dominance: Which Are the Important Variables That Influence People’s Decisions?

In addition to overall preference, psychologists may be interested in overall dominance which reveals the variable(s) which obtained the most attention from participants. Analysis 2 addresses this issue by adopting the QDominance()function. The analysis procedure is carried out by Line 112 to 148 in QmultiProtocol.R. Lines 142–148 generate a matrix of dominance weights with participants in rows and variables in columns. The result of the IOs study example is presented in Table 4.

**TABLE 4:** The variable weights for each participant of the study example.

**Participant ID**	**Size**	**Texture**	**Contour**	**Behavior**
P01	0.19	0.21	0.06	0.53
P02	0.20	0.23	0.04	0.53
P03	0.17	0.48	0.13	0.21
P04	0.14	0.27	0.23	0.36
P05	0.18	0.09	0.05	0.68
P06	0.05	0.16	0.16	0.63
P07	0.08	0.15	0.38	0.40
P08	0.18	0.49	0.04	0.30
P09	0.14	0.23	0.23	0.39
P10	0.18	0.34	0.04	0.44
P11	0.03	0.03	0.13	0.81
P12	0.02	0.00	0.24	0.74
P13	0.02	0.44	0.08	0.46
P14	0.02	0.37	0.21	0.40
P15	0.07	0.18	0.22	0.52
P16	0.44	0.15	0.13	0.28
P17	0.09	0.03	0.00	0.88
P18	0.15	0.14	0.20	0.50
Mean	0.13	0.22	0.14	0.50

**FIGURE 3:**
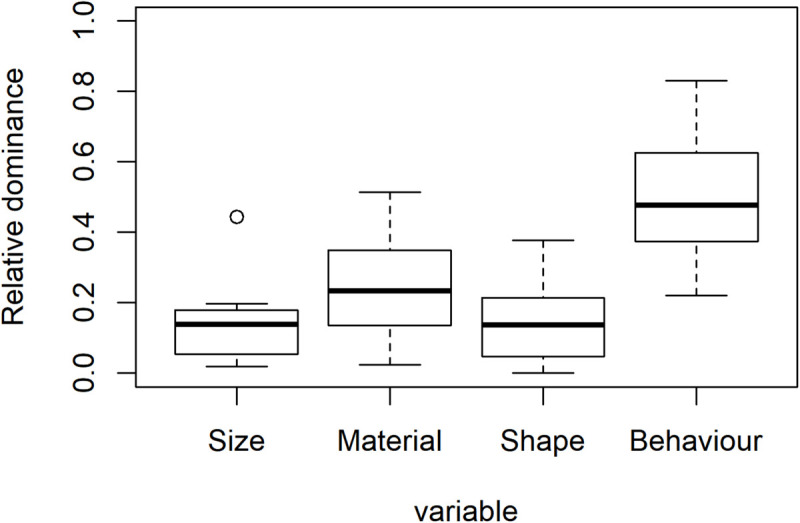
The overall dominance for the four variables of the study example.

The weights are compared by the lm()function as they distribute at an interval level. This is implemented by Lines 135–136 of the QmultiProtocol.R. The results of the IOs study example are shown in Figure 3 and Tables 5, 6. As can be seen, it seems that overall the participants took into consideration the Behavior variable more than the other variables, which suggests that the Behavior variable plays a relatively dominant role in influencing participants’ preference of IOs.

**TABLE 5:** The estimates of the linear model of overall dominance.

	***Estimate***	***t-value***	***p***
Intercept (Size)	0.13	4.04	<0.001
Texture	0.09	1.96	0.054
Contour	0.01	0.25	0.806
Behavior	0.37	8.06	<0.001

**TABLE 6:** Result of analysis of variance.

	***Df***	***Sum squares***	***Mean squares***	***F***	***p***
Variables	3	1.62	0.54	28.34	<0.001
Residuals	68	1.30	0.02		

To summarize, the analysis 1 and 2 generate different information about the decision process for all participants, namely, preference and dominance. To use the IOs study as an example, the analysis of preference suggests that, overall, participants expressed specific preferences for surface texture, shape and behavior of the IOs but not for the size. Furthermore, the analysis of dominance suggests that the Behavior variable plays the most important role in participants’ preference of IOs overall.

## Analysis 3: Individual Differences: Do People Differ in Their Decisions?

Analysis 3 consists of three steps: Q-factor analysis, variance analysis of Q-factor scores and analysis of dominance in clusters. Each analysis sheds light on a different aspect of the individual differences in people’s decision processes.

### Q Factor Analysis

Q-factor analysis groups participants together instead of items (Brown, 1980). Q-factor analysis has been used in several experimental psychology studies to examine individual differences. For example, McManus et al. (2010) found that the participants could be categorized into two clusters based on their aesthetic preferences for rectangles: those who preferred rectangles closer to a square shape, and those preferring elongated rectangles. Spehar et al. (2016) identified two clusters of participants based on their appreciation of fractal patterns: one cluster preferred images with extreme values of the spectrum slope and the other preferred intermediate slope values. Soranzo et al. (2018) found that the participants of their experiments could be categorized into two clusters based on their aesthetic preferences for interactive objects (i.e., physical artifacts that exhibit autonomous behavior when handled): one cluster based their judgments on the objects’ behavior only and the other also took into consideration the objects’ texture and shape. Apparently, experimental psychologists have been paying attention to individual differences, especially in situations where the overall preference or dominance may have been masked by individual differences that balance out the effect.

As in conventional factor analysis, principal component analysis(PCA) or centroid factor analysis is often used to analyze the Q-sorting data (Brown, 1980). The QmultiProtocol.R (Lines 153–158) employs the ‘qmethod’ (Zabala, 2014) to conduct PCA with Varimax rotation. Users can adjust the method of rotation based on their own data.

As mentioned earlier, users need to enter the number of Q-factors to extract in Line 3 of QmultiProtocol.R. The number of Q-factors is dependent on the data as well as the research topic. Watts and Stenner (2012) provided a discussion on the criteria to use to decide how many Q-factors to extract, including the Kaiser-Guttman criterion (Guttman, 1954; Kaiser, 1960, 1970), the magic number seven (Brown, 1980), the scree test (Guttman, 1954; Cattell, 1966), parallel analysis (Horn, 1965) and two (or more) significantly loading Q sorts and Humphrey’s Rule (Brown, 1980). To decide how many Q-factors to extract, users can check these criteria in the text file *Qfact.txt* generated by export.qm() function (Zabala, 2014) in Line 158 of QmultiProtocol.R. Users can test out different factor solutions to identify the one that best fits the data (see Brown, 1980; Watts and Stenner, 2012 for more details).

In the IOs study example, we compared different factor solutions with regard to the factor eigenvalues, the variance explained by factors, the number of participants loaded on each factor and the factor scores. As a result, the three-factor solution was chosen because it fits the data best and demonstrates a sound representation of the participants’ Q-sorting outcomes. The three Q-factors account for 70% of the variance. Table 7 shows the factor loadings of participants. Eight participants were significantly loaded on Q-factor 1, six on Q-factor 2 and three on Q-factor 3, which suggests that there are three clusters of participants expressing different preferences of the IOs. It is important to note that the participants are grouped on the basis of their differences in decision process which preference and dominance are intertwined. In the remaining of the Protocol these aspects are further explored.

**TABLE 7:** The Q-factor loadings of participants of the study example.

**Participant**	**Q-factor 1**	**Q-factor 2**	**Q-factor 3**
P01	0.28	0.70*	0.29
P02	0.24	0.74*	0.05
P03	−0.36	0.65*	−0.38
P04	0.58*	0.18	0.47
P05	0.47	0.61*	−0.04
P06	0.54	0.43	0.41
P07	0.16	0.80*	0.22
P08	0.82*	−0.16	0.13
P09	0.71*	0.16	0.16
P10	0.89*	0.27	−0.15
P11	0.02	−0.04	0.86*
P12	0.09	0.28	0.86*
P13	0.91*	0.16	0.02
P14	0.83*	0.26	0.05
P15	0.20	0.81*	0.37
P16	0.52*	0.28	0.10
P17	0.06	0.17	0.85*
P18	0.70*	0.62	0.12
Eigenvalue	5.4	4.1	3.1
Variance	30%	23%	17%

*Indicates significant loading.

Table 8 illustrates the factor scores of the IOs for each Q-factor. Using the object labeled LRRL (*Large*-*Rough-Round-Lighting)* as an example, participants clustered into Q-factor 1, on average (weighted average is used to calculate the factor scores of each stimuli; Watts and Stenner (2012) ranked it as slightly less preferred (−1); participants clustered into Q-factor 2, on average, ranked it as most preferred (+5) and participants clustered into Q-factor 3, on average, ranked it as slightly referred (+1). By reading these factor scores, we can gain an understanding of how different clusters of participants rank-ordered the IOs in the Q-sorting procedure.

**TABLE 8:** The Q factor scores of each IO.

**IO**	**Q-factor 1**	**Q-factor 2**	**Q-factor 3**
LRAQ	−2	0	−2
LRAV	−2	1	2
LRAL	−1	1	−1
LRAS	−4	−2	0
LRRQ	−1	2	−3
LRRV	0	4	4
LRRL	−1	5	1
LRRS	−4	−3	1
LSAQ	1	−2	−4
LSAV	1	−1	2
LSAL	4	0	2
LSAS	−1	−4	−1
LSRQ	2	−1	−2
LSRV	3	2	3
LSRL	3	3	1
LSRS	−3	−3	−1
SRAQ	0	0	−4
SRAV	0	2	3
SRAL	0	1	0
SRAS	−5	−2	0
SRRQ	0	1	−3
SRRV	0	3	5
SRRL	1	4	0
SRRS	−3	−3	0
SSAQ	3	−1	−5
SSAV	2	0	3
SSAL	5	0	1
SSAS	−3	−4	−2
SSRQ	1	−1	−3
SSRV	2	0	4
SSRL	4	3	0
SSRS	−2	−5	−1

*The first letter of IO represents the size (L = Large, S = Small); the second is the surface texture (R = Rough, S = Smooth); the third is the contour (A = Angular, R = Round); and the forth is the behavior (Q = Quiescent, V = Vibrate, L = Light, S = Sound).*

### Analysis of Variance of Q-Factor Scores for Preference

Subsequently, analysis of variance on the factor scores can be run to examine whether there is a difference in preference within variable for each cluster of participants (Brown, 1993). This is carried out from Line 160 to 171 in QmultiProtocol.R. Table 9 shows the results of the IOs study example.

**TABLE 9:** The results of analysis of variance of Q-factor scores.

		**Size**	**Texture**	**Contour**	**Behavior**
Q-factor 1	*Df*_1_, *Df*_2_	1, 30	1, 30	1, 30	3, 28
	*F*	0.48	13.01**	0.08	12.06***
	*p*	0.495	0.001	0.786	<0.001
Q-factor 2	*Df*_1_, *Df*_2_	1, 30	1, 30	1, 30	3, 28
	*F*	0.08	3.54	2.45	19.95***
	*p*	0.786	0.070	0.128	<0.001
Q-factor 3	*Df*_1_, *Df*_2_	1, 30	1, 30	1, 30	3, 28
	*F*	0.08	0.02	0.69	59.80***
	*p*	0.786	0.892	0.413	<0.001

### Analysis of Dominance in Clusters

To further explore each cluster of participants, the factor scores can be analyzed using the QDominance() function by setting the isfs parameter to True (Line 175 in QmultiProtocol.R). The result generates the dominance weights for each cluster of participants (i.e., Q-factors). It provides a straightforward overview of how the variables are weighted by each cluster of participants.

**FIGURE 4:**
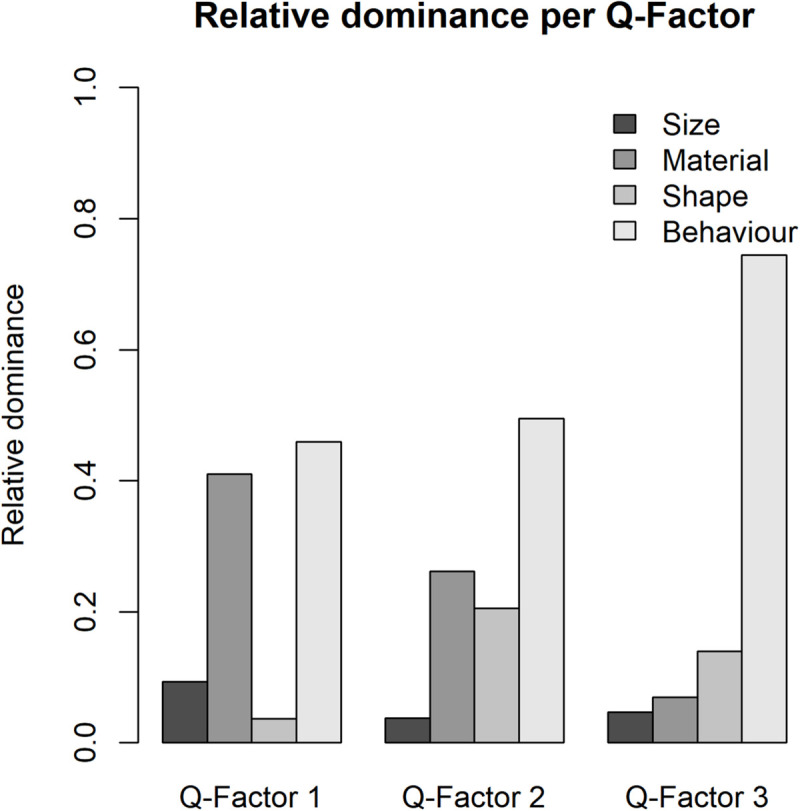
The bar chart of the dominance for each Q-factor of the study example.

As for the present IOs study example, the results of analysis of dominance in clusters are illustrated in Table 10 and Figure 4, participants in Q-factor 2 and 3 mainly paid attention to the Behavior variable; whereas participants in Q-factor 1 considered both Surface texture and Behavior variables. These dominance weights depict a straightforward picture of how each cluster of participants differs or concurs in the dominance of decision process.

**TABLE 10:** The dominance weights for each Q-factor.

	**Q-factor 1**	**Q-factor 2**	**Q-factor 3**
Size	0.093	0.038	0.049
Texture	0.410	0.248	0.024
Contour	0.037	0.210	0.146
Behavior	0.459	0.504	0.780

## Analysis 4: Interaction Between Individual Differences and Preferences: Do Different Clusters of People Prefer Different Characteristics of a Variable?

Analysis 4 focuses on the interaction between individual differences and preferences. Accordingly, QmultiProtocol.R runs a cumulative link model by the clm() function (Christensen, 2018).

**FIGURE 5:**
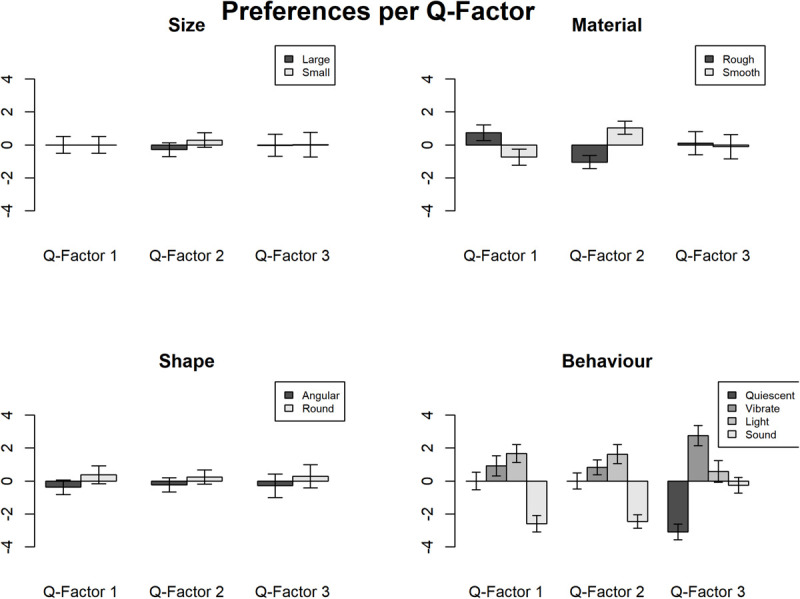
The ranks of preference of each of the three Q-factors of the study example.

Detailed estimates and coefficients of the IOs study example are presented in Tables 11, 12. Figure 5 shows these interactions in graphs. As can be seen, interesting interactions emerge when the preferences are explored as a function of individual differences. For example, participants in the Q- factor 1 expressed a preference for rough to smooth texture in whilst participants in Q-factors 2 preferred smooth to rough texture. Besides, the interaction between preference of behavior and the Q-factors shows that the participants in Q-factor 3 disliked quiescent objects whereas participants in Q-factor 1 and 2 showed a strong dislike of sounding objects.

**TABLE 11:** The variable estimates of the study example.

	***Estimate***	***Z***	***p***
Q-factor.2	−0.18	−0.37	0.710
Q-factor.3	0.02	0.04	0.968
SizeSmall	0.22	1.01	0.311
TextureSmooth	0.33	1.46	0.143
ContourRound	0.26	1.14	0.253
BehaviourVibrate	1.57	4.13	<0.001
BehaviourLight	1.63	3.52	<0.001
BehaviourSound	−1.28	−3.32	<0.001
Q-factor.2:SizeSmall	−0.07	−0.20	0.84
Q-factor.3:SizeSmall	0.10	0.22	0.82
Q-factor.2: TextureSmooth	−0.22	−0.63	0.53
Q-factor.3: TextureSmooth	0.44	1.01	0.31
Q-factor.2: ContourRound	0.61	1.78	0.07
Q-factor.3: ContourRound	0.03	0.06	0.95
Q-factor.2: BehaviourVibrate	0.81	1.32	0.19
Q-factor.3: BehaviourVibrate	−0.19	−0.29	0.77
Q-factor.2: BehaviourLight	0.15	0.28	0.78
Q-factor.3: BehaviourLight	0.30	0.47	0.64
Q-factor.2: BehaviourSound	0.28	0.46	0.64
Q-factor.3: BehaviourSound	−0.70	−1.04	0.30

**TABLE 12:** Result of Wald chi-square test for the Preference per Q-factor interactions.

**Variable**	***df***	***Chi*-square**	***p***
Factor * Size	2	0.13	0.935
Factor * Texture	2	1.98	0.372
Factor * Contour	2	3.52	0.172
Factor * Behavior	6	5.32	0.503

## Analysis 5: Interaction Between Individual Differences and Dominance: Are Different Clusters of People Driven by Different Variables?

Analysis 5 focuses on the interaction between individual difference and dominance. It is conducted by simply adding a between-subjects variable indicating the Q-factor of each participant to the dominance weights obtained by QDominance(). A Linear model with interaction between individual differences and dominance is conducted subsequently as shown in QmultiProtocol.R (Lines 304–332).

**FIGURE 6:**
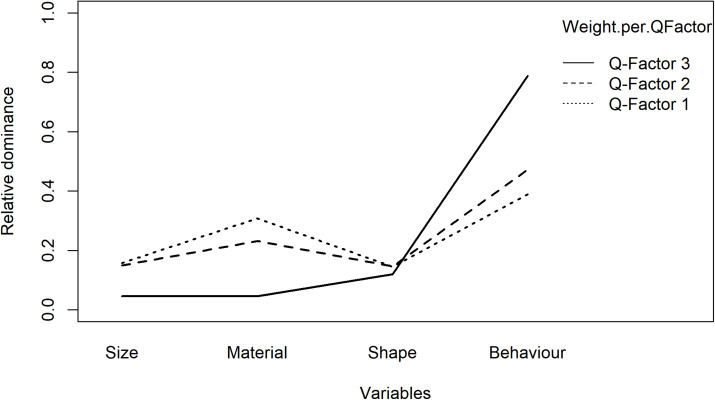
The interaction between the dominance and the Q-factors of the study example.

In the present IOs study example, the results are shown in Figure 6 and Tables 13, 14. As can be inferred from the results, participants in Q-factor 3 were considerably different from participants in Q-factor 1 and 2 in terms of the variables they paid attention to when choosing an IO.

**TABLE 13:** Estimates of the linear model of the interaction between individual differences and dominance.

	***Estimate***	***t-value***	***p***
Intercept (Size)	0.16	4.06	<0.001
Texture	0.14	2.61	0.011
Contour	−0.01	−0.24	0.815
Behavior	0.23	4.22	<0.001
Factor2	−0.01	−0.14	0.892
Factor3	−0.11	−1.48	0.146
Texture*Factor2	−0.07	−0.84	0.406
Contour*Factor2	0.01	0.12	0.908
Behavior*Factor2	0.09	1.12	0.273
Texture*Factor3	−0.17	−1.63	0.109
Contour*Factor3	0.09	0.82	0.418
Behavior*Factor3	0.53	4.99	<0.001

**TABLE 14:** Result of analysis of variance.

	***Df***	***Sum squares***	***Mean squares***	***F***	***p***
Variables*Factor	6	0.59	0.10	8.02	<0.001
Residuals	56	0.68	0.01		

## Validation of the Method

As mentioned, the aesthetics of the IOs has been measured in Soranzo et al. (2018). Two phases were carried out in the study, a qualitative phase to individuate the aesthetics dimensions involved and a quantitative phase where participants were requested to rate each IO on a Likert scale for each of the dimensions emerged in the first study. In the quantitative phase, over 600 participants were needed and one variable was manipulated between subjects to minimise participants’ fatigue. Results obtained with the new method are consistent with those obtained with the traditional method in terms of the preferences: favored aesthetics features are vibration (in line with Carbon and Jakesch, 2013), roughness (in line with Jehoel et al., 2005) and roundness (in line with Bertamini et al., 2016). As seen, with only 18 participants it was possible, in this case, to get comparable results as with over 600. In addition, being the task more enjoyable and quicker for the participants it was possible to run all the variables within-participants allowing for more reliable results.

Moreover, clear advantages of the new procedure are the following:

Dominance emerged more clearly. With traditional Likert scale methods, the dominance of a variable could be inferred only indirectly, whilst this procedure explicitly reveals this feature.

Q-factors are clearer. Both analyses showed similar factorial solutions; however, with the new method it was possible to compare both preference and dominance for the different clusters of participants.

The qualitative data were obtained directly, from the same participants that ranked the IOs, speeding up the procedure. Such qualitative data generate in-depth perspective on how participants make aesthetic decisions during experiments.

## Limitations

There are a few limitations in applying this method. Firstly, it requires a relatively large number of stimuli for Q-sorting to ensure the validity and credibility of statistics tests. Secondly, given that all the stimuli need to be presented at the same time for participants to choose from, it may turn out to be tricky to conduct online Q-sorting or use digital stimuli because they may not all fit in the screen. Finally, as Q methodology is not yet widely used in experimental pscyhology, the researcher may require more effort to describe the method to disseminate their finding.

## Conclusion

This project extends the application of Q-methodology to experimental psychology by incorporating ‘QDominance()’ to analyze data varying in multiple variables and to interpret the dominance. The combination of Q-methodology with ‘QDominance()’ has numerous methodological advantages and can bring about new insights into the field of experimental psychology.

We provide a protocol of five analyses which are adaptable to a wide range of psychological experiments. While this guide adopts a specific experiment on aesthetics study as the example, the same procedure can be used in various aesthetics contexts such as preferences or judgments of similarity/dissimilarity to a model. By following the protocol or picking and choosing the analysis as needed, experimental psychologists can address a variety of research questions. An R script to run the five analyses is provided which can be readily used with very little further programming.

The advantages of this method are evident when compared with more traditional methods and can be summarized as follow: quicker and more enjoyable for the participants (giving rise to more reliable results); more straightforward demonstration of clearer Q-factors, clearer and direct comparison between preference and dominance among the Q-factors.

In addition, while the protocol mainly focuses on further developing the quantitative analysis procedure of Q-methodology, the qualitative analysis of post-sorting interview data should not be neglected. Experimental psychologists can make the most of the methodological advantages of Q-methodology by applying this protocol together with proper qualitative analysis of the post-sorting interview in Q.

## Data Availability Statement

The original contributions presented in the study are included in the article/Supplementary Material, and here: https://osf.io/pzvfb. Further inquiries can be directed to the corresponding author.

## Ethics Statement

The studies involving human participants were reviewed and approved by Sheffield Hallam University ethics committee. The patients/participants provided their written informed consent to participate in this study.

## Author Contributions

AS developed the R script and the Dominance function, and wrote the manuscript. JG applied the method to a practical example and wrote the manuscript. All authors contributed to the article and approved the submitted version.

## Conflict of Interest

The authors declare that the research was conducted in the absence of any commercial or financial relationships that could be construed as a potential conflict of interest. The reviewer MB declared a past co-authorship with one of the authors, AS, to the handling editor.

## Footnotes

1 There is a debate about whether the use of free distributions is acceptable in Q methodology. Interested readers can refer to Watts and Stenner, 2012, p.77.

2 https://osf.io/pzvfb/
