# Supplementary material for: Applying Q-Methodology to Investigate People’ Preferences for Multivariate Stimuli
Source: Front Psychol. 2020 Dec 10;11:556509. doi: 10.3389/fpsyg.2020.556509 (PMC7758491; doi:10.3389/fpsyg.2020.556509)
Supplement: Supplementary file 1 [file Data_Sheet_1.ZIP › AppQ-methodologytoExpPsy - sup mat/Step-by-step Tutorial.pdf]

Tutorial: Using QmultiProtocol

This tutorial accompanies the paper *"Applying Q-methodology to investigate people' preferences for multivariate stimuli"* by Gao & Soranzo (2020). Please refer to the paper for details.

Step-by-step tutorial:

1. **Download the full project here:** https://osf.io/pzvfb/

2. **Put the following files in your working directory**

    - Data.csv (example of data)
    - Variables.csv (example of variables)
    - QDominance.R (function to calculate the dominance and preference)
    - QmultiProtocol.R

The remaining of the tutorial refers to the QmultiProtocol.R

3. **Set your working directory in R:**

```
setwd('the working directory you created')
```
(line 3)

4. **Set the number of factors you decide to extract for the Q factor analysis** (line 5)

```
nfactors<-
```

[After the first run of the QmultiProtocol.R the user will find a file named *Qfact.txt* in the same working directory as per line 1. This file contains useful information to decide how many Q-factors to select. This can be therefore changed in successive runs of the protocol.]

5. **Install the packages that are required for this analysis** (lines 7-12)

```
if(!require(data.table)){install.packages("data.table")
  library(data.table)}
if(!require(ordinal)){install.packages("ordinal")
```

```
  library(ordinal)}
if(!require(qmethod)){install.packages("qmethod")
  (library (qmethod))}
```

6. **Calculate the 'dominance' by using the 'QDominance' function:** (lines 14-19)

```
source ("QDominance.R")
data <- read.csv('data.csv', header = F)
variables <- read.csv('variables.csv', header = T)
warning(-1)
if (nfactors < 2)
  warning("Only one factor selected. No Means of Analysis 3, 4 and
 5 are conducted")
```

7. **Examine the overall preference: (*i.e.,* Address the research question: Which are the overall preferred characteristics of each variable?)**
   Use the script of the 'Analysis 1' in the *QmultiProtocol.R* (line 24-110)

8. **Examine the overall dominance: (*i.e.,* Address the research question: Which are the important variables that influence people's decisions?)**
   Use the script of the 'Analysis 2' in the QmultiProtocol.R (line 117-149)

9. **Examine the individual differences: (*i.e.,* Address the research question: Do people differ in their decisions?)**
   Use the script of the 'Analysis 3' in the QmultiProtocol.R (line 155-191)

10. **Examine the interaction between individual differences and preferences: (*i.e.,* Address the research question: Do different clusters of people prefer different characteristics of a variable?)**
    Use the script of the 'Analysis 4' in the QmultiProtocol.R (line 197-303)

11. **Examine the interaction between individual differences and dominance: (*i.e.,* Address the research question: Are different clusters of people driven by different variables?)**

    Use the script of the 'Analysis 5' in the QmultiProtocol.R (line 309-333)
